# Cholangioscopy-guided lithotripsy vs. conventional therapy for complex bile duct stones: a systematic review and meta-analysis

**DOI:** 10.1590/0102-672020190001e1491

**Published:** 2020-06-26

**Authors:** Facundo GALETTI, Diogo Turiani Hourneaux de MOURA, Igor Braga RIBEIRO, Mateus Pereira FUNARI, Martin CORONEL, Amit H. SACHDE, Vitor Ottoboni BRUNALDI, Tomazo Prince FRANZINI, Wanderley Marques BERNARDO, Eduardo Guimarães Hourneaux de MOURA

**Affiliations:** 1 Unidade de Endoscopia Gastrointestinal, Hospital das Clínicas, Universidade de São Paulo, São Paulo, SP, Brasil; 2 Divisão de Gastroenterologia, Hepatologia e Endoscopia, Brigham and Women´s Hospital, Harvard Medical School, Boston, Massachusetts, EUA

**Keywords:** Endoscopic retrograde cholangiopancreatography, ERCP, Lithotripsy, Choledocholithiasis, Systematic review, Meta-analysis, Colangiopancreatografia retrógrada endoscópica, CPRE, Litotripsia, Coledocolitíase, Revisão sistemática, Metanálise

## Abstract

**Introduction:**

Endoscopic removal of common bile duct stones has a high success rate ranging from 85% to 95%. Bile duct stones >15 mm are difficult and frequently require lithotripsy. Peroral cholangioscopy (POC) allows lithotripsy with similar success rates.

**Aim:**

To determine the efficacy and safety of cholangioscopy-guided lithotripsy used in the treatment of difficult to remove bile duct stones vs. conventional therapy.

**Methods:**

Search was based in Medline, Embase, Cochrane Central, Lilacs/Bireme. Studies enrolling patients referred for the removal of difficult bile duct stones via POC were considered eligible. Two analyses were carried out separately, one included randomized controlled trials (RCTs) and another observational studies.

**Results:**

Forty-six studies were selected (3 RTC and 43 observational). In the analysis there was no statistical significant difference between successful endoscopic clearance (RD=-0.02 CI: -0.17, 0.12/I²=0%), mean fluoroscopy time (MD=-0.14 CI -1.60, 1.32/I²=21%) and adverse events rates (RD=-0.06 CI: -0.14, 0.02/I²=0%), by contrast, the mean procedure time favored conventional therapy with statistical significance (MD=27.89 CI: 16.68, 39.10/I²=0%). In observational studies, the successful endoscopic clearance rate was 88.29% (CI95: 86.9%-90.7%), the first session successful endoscopic clearance rate was 72.7 % (CI95: 69.9%-75.3%), the mean procedure time was 47.50±6 min for session and the number of sessions to clear bile duct was 1.5±0.18. The adverse event rate was 8.7% (CI95: 7%-10.9%).

**Conclusions:**

For complex common bile duct stones, cholangioscopy-guided lithotripsy has a success rate that is similar to traditional ERCP techniques in terms of therapeutic success, adverse event rate and means fluoroscopy time. Conventional ERCP methods have a shorter mean procedure time.

## RESUMO

**Introdução:**

A remoção endoscópica das litíases do ducto biliar comum tem alta taxa de sucesso variando de 85% a 95%. Litíases do ducto biliar >15 mm são difíceis e frequentemente requerem litotripsia. A colangioscopia peroral permite litotripsia com taxas de sucesso semelhantes.

**Objetivo:**

Determinar a eficácia e segurança da litotripsia guiada por colangioscopia no tratamento de litíases biliares difíceis em comparação **à** terapias convencionais guiadas por colangiopancreatografia retrógrada endoscópica.

**Método:**

Pesquisa na Medline, Embase, Cochrane Central, Lilacs/Bireme de estudos avaliando a eficácia da colangioscopia na remoção de cálculos biliares difíceis. Duas análises foram realizadas separadamente, uma incluiu ensaios clínicos randomizados (ECR) e outros estudos observacionais.

**Resultados:**

Quarenta e seis estudos foram selecionados (3 ECR e 43 observacionais). Na análise, não houve diferença estatisticamente significativa na taxa de extração litiásica total (RD=-0,02 IC: -0,17,0,12/I²=0%), tempo médio de fluoroscopia (MD=-0,14 CI -1,60, 1,32/I²=21%) e na taxa de eventos adversos (RD=-0,06 IC: -0,14, 0,02/I²=0%).Por outro lado, o tempo médio do procedimento favoreceu terapêuticas convencionais guiadas por CPRE com significância estatística (MD=27,89 IC: 16,68, 39,10/I²=0%). Nos estudos observacionais, a taxa do tratamento completo por endoscopia foi de 88,29% (IC95: 86,9% a 90,7%), a taxa de sucesso na primeira sessão foi de 72,7% (IC95: 69,9% a 75,3%), o tempo médio do procedimento foi de 47,50±6 min por sessão e o número de procedimentos necessários para remoção total da litíase foi de 1,5±0,18. A taxa de eventos adversos foi de 8,7% (IC95: 7% a 10,9%), com 0,5% considerado como severo.

**Conclusão:**

Para litíases biliares difíceis, a litotripsia guiada por colangioscopia tem taxa de sucesso semelhante às terapêuticas convencionais guiadas CPRE em termos de sucesso terapêutico, taxa de eventos adversos e tempo de fluoroscopia. As terapêuticas convencionais guiadas por CPRE têm tempo médio de procedimento menor.

## INTRODUCTION

This is a systematic review with a meta-analysis that evaluates the use of peroral therapeutic cholangioscopy in the management of in difficult bile duct stones and is the first in the literature to include and perform meta-analyzis of randomized clinical trials. This is also the first systematic review to include randomized controlled trials (RCTs) comparing peroral cholangioscopy (POC) vs. conventional endoscopic retrograde cholangiopancreatography (ERCP) therapies used in the management of difficult bile duct stones

Approximately 85-95% of gallstones in the bile duct can be managed with ERCP techniques, such as performing a sphincterotomy or using papillary balloon dilatation, extractor balloon, basket or mechanical lithotripter^67^ **.** Although successful most of the time, certain cases of biliary lithiasis are difficult or impossible to manage with conventional techniques with a failure rate as high as 10-15% reported in some studies^14^ ^,^ ^52^ ^,^ ^57^ ^,^ ^61^ ^,^ ^65^ **.**

Over the last decade, technological advances have made POC a more accessible alternative technique that can be used in the management of complex bile duct stones, especially after failure of the initial ERCP. Classically, there are three types of cholangioscopy: cholangioscopic dual operator system (“mother-daughter” system); direct with an ultra-thin endoscope (ultraslim endoscope); and the cholangioscopic system with a single operator catheter (peroral acronym cholangioscopy, POC)^33^ **.**

POC was most recently developed and has been extensively studied and marketed. However, the actual efficacy and adequate indication for it in the management of difficult bile duct stones is not yet clear. Systematic reviews have been published on the subject; however, there was not include all the RTCs available in the literature and when performed the meta-analysis they unified RTCs and observational studies, which diminishes the strength of the evidence.

The primary objective of this systematic review and meta-analysis is to compare the therapeutic success of POC and standard ERCP endoscopic methods in the management of difficult bile duct stones. Secondary objectives include comparing the overall adverse event rate, mean procedure time and the mean fluoroscopy time of these techniques in the management of difficult bile duct stones.

## METHODS

This study was approved by the Research Ethics Committee of the University of São Paulo, School of Medicine, Hospital das Clínicas (registration number 239/19). It followed the Preferred Reporting Items for Systematic Reviews and Meta-Analyses (PRISMA) guidelines^44^ **.** It was registered in the international PROSPERO database (number CRD42018109952).

### Definitions

The definition of complex bile duct stones included factors such as stone size (greater than 15 mm), disproportion of the stone within the common distal bile duct, patients with altered gastrointestinal anatomy, biliary strictures, multiple stones, barrel-shaped stones and difficult access sites (intrahepatic lithiasis, Mirizzi I syndrome). Therapeutic success was defined as complete removal of the stone of the common bile duct and clearance of the same.

### Search strategy

We searched in Medline (Pubmed), Embase, Cochrane Central and Lilacs/Bireme (until February 2019) for the studies and assessed the efficacy of POC for the removal of complex biliary stones. The terms used in Medline were: (choledocholithiasis OR stone* OR calculus OR lithiasis OR calculi) AND (cholangiopancreatoscopy OR choledochoscopy OR pancreatocholangioscopy OR cholangioscopic OR lithotrips*). Simpler search strategies were used for Embase, Cochrane Central, and Lilacs/Bireme databases. The search was restricted to human studies with no language or date of publication restriction in peer-reviewed journals. Two authors (FG and IBR) independently screened each of the potential manuscript and abstracts titles in the primary search to exclude studies that did not address the research question of interest, based on pre-specified inclusion and exclusion criteria. The full texts of the remaining articles were examined to determine whether they contained relevant information. Areas of disagreement or uncertainty in article selection were resolved by consensus and in discussion with a coauthor (EGHM). Conference proceedings, which did not undergo peer-review, were excluded from our analysis. We attempted to contact the corresponding authors to provide additional information on trials if required.

### Eligibility

RCTs, observational cohort studies and case series which met inclusion criteria were considered eligible. Conference abstracts were also included if they met the eligibility criteria listed below. The eligibility criteria were based on study participants, intervention type, comparison type, and outcomes (PICO): (P) participants: patients with complex bile duct stones; (I) intervention types: cholangioscopy-guided laser lithotripsy (LL) or electrohydraulic lithotripsy (EHL); (C) comparison types: conventional therapy; and (O) outcomes measures: successful stone clearance, adverse event rate, mean procedure time, mean fluoroscopy time, successful stone clearance after one attempt, and the total number of sessions necessary to extract biliary stones of POC in the management of difficult bile duct stones. Exclusion criteria were: case reports, reviews, letter to authors or editors, animal studies, studies evaluating pancreatoscopy, studies with patients with malignant pancreatobiliary disease, surgical and/or anatomical alterations of the gastrointestinal or biliopancreatic tract and studies evaluating percutaneous cholangioscopy.

### Data extraction and quality assessment

We conducted the analysis in two different forms: the first one using only randomized trials, for comparative analysis; and the second with observational studies, for non-comparative analysis. Each study was analyzed for: publication year; study design (RCT, prospective or retrospective); setting (single center or multicenter); type of cholangioscopy; sample size; intervention (LL or EHL); comparison (conventional therapy), endoscopic stone clearance, adverse events; mean procedure time and mean fluoroscopy time.

For observational studies additional data included the following: first session success rate and number of sessions to clear the bile duct. This data was extracted and documented on a standardized data form by at least two authors independently (FG, VOB). 

The quality of each clinical trial was classified according to the risk for bias and based upon: the question to be investigated, the use of a correct randomization protocol, an adequate subject allocation, the importance of blinding, patient losses in each study, each prognostic factor, outcome reporting and analysis by intention to treat or by protocol^22^. In addition, the Jadad scale was used to independently assess the methodological quality of each clinical trial^26^. The quality of the evidence of the included studies was evaluated according to GRADE standards using the GRADEpro Guideline Development Tool software^21^.

### Statistical analysis

Absolute numbers, mean and standard deviations were used for quantitative data analysis. For studies that did not determine standard deviations, the standard error and confidence interval were estimated using mathematical formulas^72^.

Comprehensive Meta-Analysis (Englewood, NJ) and Review Manager version 5.3.5 (RevMan 5.3-The Cochrane Collaboration, The Nordic Cochrane Centre, Copenhagen, Denmark) were used to conduct the meta-analysis and develop the forest plot graphs. For continuous variables, the mean difference between the groups was calculated using the mean, standard deviation, and sample size of each group. For dichotomous variables, the risk difference was determined by calculating the number of events and sample size of each group. Heterogeneity was evaluated using the chi-square test (χ^2^) and funnel plot analysis was performed to identify outlying studies. Heterogeneity values of greater than 50% were considered high. In cases in which it was impossible to correct the heterogeneity by excluding the outlier, a fixed analysis model was changed to a random model.

### Additional analyses

In order to compare the two types of cholangioscopic lithotripsy, a subgroup analysis in observational studies using only cholangioscopy was performed comparing LL and EHL successful stone clearance means in the complete removal of complex bile duct stones. The mean effect measure was calculated for the subgroups of EHL vs. LL, considering the mean of each study with the size of its sample. The total means were then compared using Student’s t-test. 

## RESULTS

**FIGURE 1:**
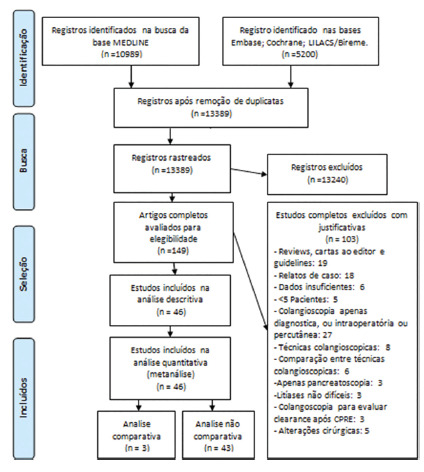
Flow diagram of the data extraction methodology

A total of 16,189 records were identified in the initial search. After removal of duplicates, 13,389 records were reviewed. After title/abstract assessment, 149 articles were selected for full evaluation. After individual review, 46 studies satisfied the inclusion and exclusion criteria and were included within the study (Figure 1).

Among the 46 studies selected for the quantitative analysis, three of them were RCTs^4^ ^,^ ^10^ ^,^ ^18^and the others were observational studies^1-3,5-9,11,12,16,17,23-25,27,29,30,32,34,35,37-40,42,43,46-48,50,51,53,55,58-60,63,66,68-70,73.^

**FIGURE 2:**
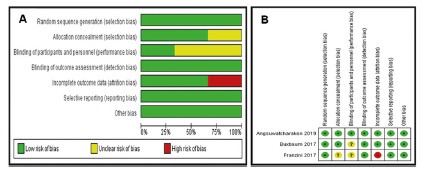
A) Summary of risk of bias of included RCT’s; B) summary of risk of bias of included RCT’s

**FIGURE 3:**
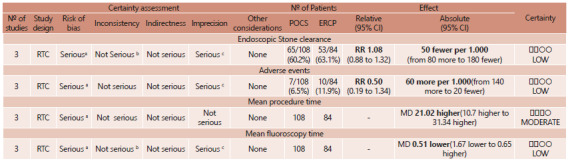
Evaluation of evidence quality of included RCTs. GRADE system Explanations: A) lack of blinding in the trials: one of the authors did not perform intention-to-treat analysis; B) wide heterogeneity between the studies, but explained; C) power for randomized clinical trials <80 %.

**FIGURE 4:**
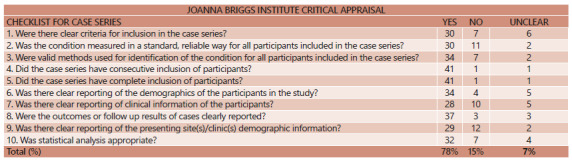
Joanna Briggs Institute critical appraisal checklist for case series

We conducted the analysis in two different forms: the first one using only randomized trials, for comparative analysis; and the second with observational studies, for non-comparative analysis. Figure 2 A-B shows the detailed bias and quality analysis of the randomized trials. In short, all studies received a Jadad score of >3, indicating adequate study quality. The quality of the obtained data of the randomized trials was assessed using the GRADE methodology based on the type of evaluated outcome (Figure 3). Details of the risk of bias and quality assessment of the observational studies are shown in Figure 4.

CI=confidence interval; RR=risk ratio; MD=mean difference.

### Comparative meta-analysis of RTCs

#### Descriptive analysis

The three RCTs compared the successful stone clearance of cholangioscopy-guided lithotripsy versus ERCP-guided endoscopic therapies in the treatment of difficult biliary stones. All the articles used were published in English and in full text. The total sample size in this analysis was one hundred and ninety-two patients. 

**FIGURE 5:**
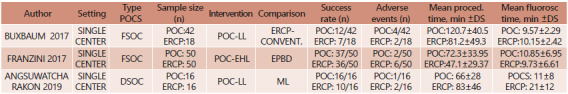
Characteristics of selected randomized controlled trials

In one group, cholangioscopic therapies were performed through EHL or LL; in the other group (control), endoscopic therapies were guided by ERCP and included sphincterotomy, papillary dilatation with balloon dilator, mechanical lithotripsy, extractor balloon and basket. The complete data characteristics are described in Figure 5.

POC=peroral cholangioscopy; ERCP=endoscopy retrograde cholangiopancreatography; DSOC=digital single-operator cholangioscopy; FSOC=fiberoptic single-operator cholangioscopy; POCS-LL=peroral cholangioscopy-guided lithotripsy; POCS-EHL=peroral cholangioscopy-guided electrohydraulic lithotripsy; ERCP-CONVENTIONAL=ERCP-guided conventional therapy; EPBD=endoscopic papillary balloon dilation; ML=mechanical lithotripsy

## Quantitative analysis (meta-analysis)

### Successful endoscopic clearance (therapeutic success)

**FIGURE 6:**
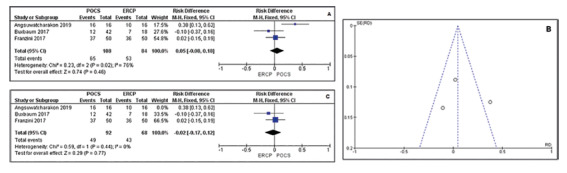
A) Forest plot of successful endoscopic clearance showed high heterogeneity; B) funnel plot of successful endoscopic clearance with outlier; C) forest plot of successful endoscopic clearance after excluding outlier study.

Three studies totaling 192 patients (108 in the POCS group and 84 in the ERCP group) compared this outcome. The meta-analysis showed no statistical difference between the techniques and showed high heterogeneity between studies (RD=0.05 IC: -0.08, 0.18 / I²= 76%). In the funnel plot analyses an outlier study was identified^4^. After exclusion of this study there was a drop in heterogeneity; however, no significant difference was observed between ERCP and cholangioscopy (RD= -0.02CI: -0.17, 0.12 /I²= 0%, Figure 6 A, B and C). 

In the analysis by the GRADE, this outcome presented a low quality of evidence since one of the studies was not double-blinded, did not perform the data analysis by intention to treat and was imprecise since the power was less than 80% due to the fact that there was no significant difference between the study groups.

### Mean procedure time

All three RCTs analyzed this outcome. The mean time of procedure was significantly lower in the ERCP group but there was high heterogeneity within the analysis (MD= 21.02 CI: 10.70, 31.34/ I²= 81%). We were able to identify the outlier study^4^ in the funnel graph analysis. After excluding this study there was a drop in heterogeneity and a significant difference was observed between ERCP and cholangioscopy (MD=27.89 CI: 16.68, 39.10/ I²=0%).

In the analysis by the GRADE, this outcome presented a moderate quality of evidence since one of the studies was not double-blinded and did not perform the data analysis by intention to treat. However, power was higher than 80% with statistical significance.

### Mean fluoroscopy time

All three randomized studies analyzed this outcome and the meta-analysis demonstrated a similar mean fluoroscopy time between techniques but with high heterogeneity (MD= -0.51CI: -1.67, 0.65/I²=76). After evaluation of the funnel plot, the Angsuwatcharakonet et al^4^ study was identified as outlier and removed from the evaluation. 

The new analysis showed low heterogeneity and no significant difference remained (MD=-0.14CI: -1.60, 1.32/I²=21%). In the GRADE analysis, this outcome presented low quality of evidence, for the same reasons as the first outcome analyzed.

### Adverse events

All three articles reported the absolute number of adverse events and totaled seven in the POCS group and 10 in the ERCP group and did not generate a statistically significant difference. The analysis showed low heterogeneity between studies (RD= -0.06 IC: -0.14, 0.02/I²= 0%). 

**FIGURE 7:**
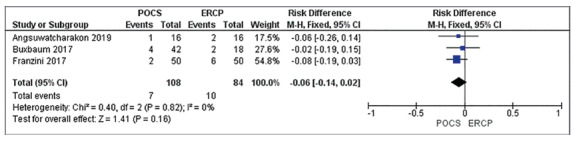
Forest plot of adverse events in RCT studies

In the GRADE analysis, this outcome presented a low quality of evidence for the same reasons given for the successful endoscopic clearance (Figure 7).

## Meta-analysis of observational studies

### Descriptive analysis

The 43 studies are retrospective or prospective case series analyzing the efficacy and safety of cholangioscopy in the treatment of difficult biliary stones. All articles were published in English and in full text. We included studies that included only patients with proposed cholangioscopy-guided lithotripsy (electrohydraulic or laser). The total number of patients studied in this analysis was 1638. 

The majority of patients had a history significant for failure to remove stones on prior ERCP attempt. In this group, five outcomes could be analyzed: successful endoscopic clearance, first session success rate, mean procedure time, number of sessions needed to clear the bile duct, and adverse event rate. The mean effect measure was calculated for the subgroups of EHL vs. LL, considering the mean of each study with the size of its sample. 

## Quantitative analysis (meta-analysis)

### Successful endoscopic clearance

**FIGURE 8:**
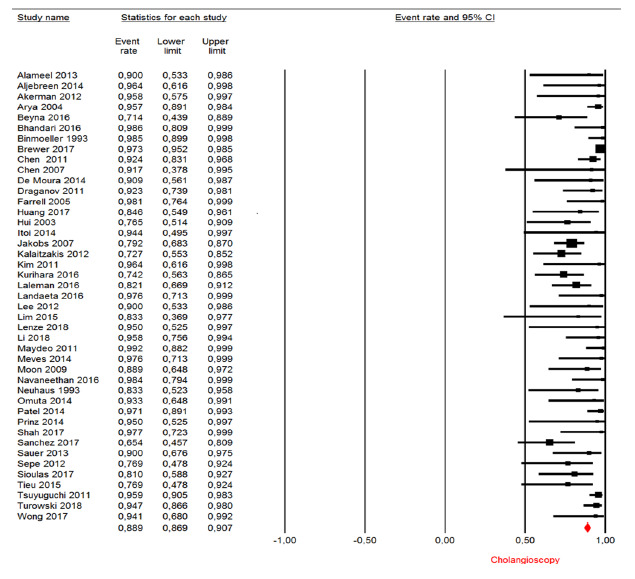
Endoscopic stone clearance: forest plot of successful endoscopic clearance rate in cholangioscopy-guided lithotripsy in observational studies

The successful endoscopic clearance rate was analyzed in 43 studies included in the qualitative analysis, totaling 1638 patients. The mean clearance rate was 88.29% (CI95: 86.9 %-90.7%, Figure 8).

### First session success rate

The rate of successful endoscopic clearance in the first cholangioscopy session was reported in 25 articles, totaling 1166 patients. The mean clearance rate was 72.7% in cases with difficult bile duct stones (CI95: 69.9%-75.3%).

### Mean procedure time to clear the bile duct

The mean procedure time of cholangioscopy-guided lithotripsy was evaluated in a total of 13 studies totaling 754 patients. On average, 47.50±6 min per session was required. 

### Number of sessions to clear the bile duct

The number of procedures required to effectively remove all difficult bile duct stones was 1.5±0.18. Twenty-four articles with a total of 1166 patients reported this outcome.

### Adverse events

**FIGURE 9:**
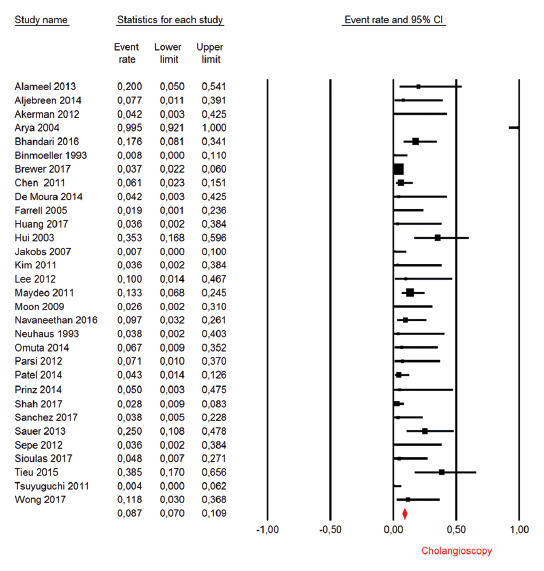
Adverse effects: forest plot of adverse events rate in cholangioscopy-guided lithotripsy in observational studies

The percentage of adverse events in the treatment of difficult bile ductstones was reported in 31 observational studies, totaling 1328 patients. The mean rate of adverse events reported was 8.7% (IC95: 7%-10.9%, Figure 9), with 0.5% of severe adverse events (assessed by ASGE lexicon, Cotton et al., 2010)^15^.

## Subgroup analysis

### EHL vs. LL

**FIGURE 10:**
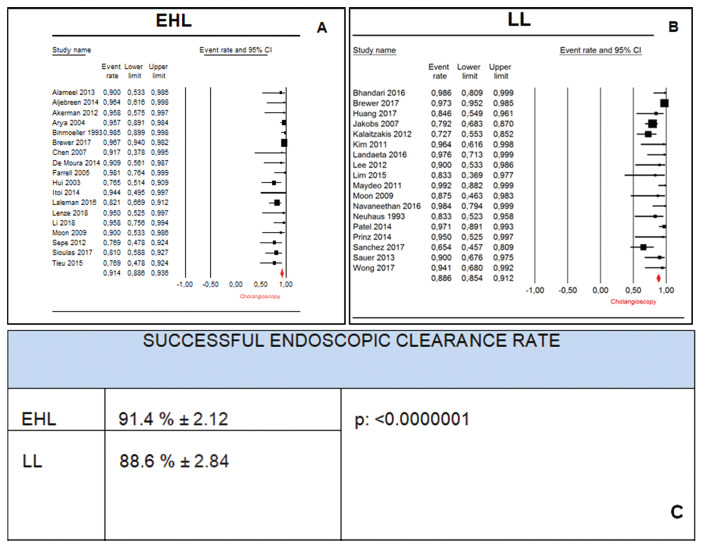
A) Successful endoscopic clearance rate using EHL; B) successful endoscopic clearance rate using LL; C) difference with statistical significance (Student’s t-test)

We compared the final mean of the observational studies that used electrohydraulic lithotripsy vs. laser lithotripsy for successful endoscopic clearance rate. The successful endoscopic clearance using only EHL was reported in 18 studies, totaling 694 patients, with a mean successful endoscopic clearance rate of 91.4% (IC95: 88.6% - 93.6%), while those who employed laser lithotripsy rate were reported in 18 articles, totaling 554 patients, with a final mean of 88.6%(IC95: 85.4%-91.2%). The final means were then compared using Student’s t-test, with statistical significance (p<0.0000001). (Figure 10 A, B and C)

## DISCUSSION

This is the first systematic review to include and perform meta-analysis of RCTs comparing POC-guided lithotripsy vs. conventional ERCP therapies in the treatment of difficult to treat bile duct stones.

There is one recent systematic review and meta-analysis regarding POC-guided lithotripsy in the management of complex biliary stones^28^. However, this study has several limitations. Jin et al.^28^ did not include all available RCTs studies and in their analyses, they performed the meta-analysis by blending data from the observational studies with the RCTs which lowered the level of evidence in the study^28^.

Individually, the RCTs available in the literature have small sample sizes, which limit the generalizability of their results^4^ ^,^ ^10^ ^,^ ^18^. Thus, this meta-analysis, which aggregates these studies, significantly improves the quality of evidence available for clinical decision making. Additionally, due to the small sample of RCTs, we chose to retrieve all the data from observational studies in order to define with greater certainty in a secondary analysis, the absolute values of the POC-associated outcomes.

There was no statistical difference in the endoscopic stone clearance rate of the complex bile duct stones treated with cholangioscopy vs. conventional therapies guided by ERCP. Additionally, adverse events and fluoroscopy time were also similar. The only statistically significant difference was in relation to the procedure time, which favors conventional ERCP. This information is in agreement with clinical practice, since the use of cholangioscopy implies the addition of another procedure to an ERCP already in progress and may increasing the costs. This data may prompt future studies of cost or cost-effectiveness.

After analysis of the three randomized studies, we found a high heterogeneity that we corrected for after we excluded an outlier study. The divergent study was published by Angsuwatcharakonet et al.^4^ and widely favored cholangioscopy.

A few differences between this study and the two other studies may explain the divergence. First, the digital version of the single operator cholangioscopy system introduced in February 2015 (DSOC, SpyGlass DS) was used instead of the previous version of fiber optics by single operator (FSOC, Legacy SpyGlass) that was used in the other two clinical trials. Shah et al. demonstrated in a controlled study that the digital version significantly improved the quality of the image compared to the previous system, possibly enhancing diagnostic and therapeutic capabilities^62^. The new device features a tapered insertion point, digital image, wider field of view and a larger working channel. Some retrospective cohorts have also demonstrated the superiority of the new POC version over the former^48^ ^,^ ^63^. This technological improvement potentially increases the chance of success in the cholangioscopy group of the Angsuwatcharakon et al. study, making the chance of success in the ERCP group significantly different^4^ **.**

Another feature of the outlier study was that all the patients that were evaluated had a previous ERCP attempt and the use of papillary large balloon dilation to remove a stone was not successful. In the studies of Franzini et al^18^. and Buxbaum et al^10^, a significant number of patients did not have ERCP prior to inclusion and cholangioscopy was used as primary therapy for difficult biliary lithiasis^,^ **.** This may explain the increased success rate in the ERCP group in these two studies, decreasing the difference compared with the cholangioscopy group. Perhaps if all three RCTs included only patients with previous ERCP attempts and used the new version of POCS, cholangioscopy would be favored in this meta-analysis. These differences could explain the divergence of the results pointed out by Angsuwatcharakon et al^4^.

Buxbaum et al. in their concluding remarks pointed out that patients with a previously failed ERCP are less likely to have biliary clearance in general^10^. In a subgroup analysis, they demonstrated a 36% increase in the success rate in patients with prior ERCP failure who were randomly assigned to the cholangioscopy arm when compared to those who went for conventional therapy. In contrast, cholangioscopy seems less likely to provide significant benefits and may even increase the time of the procedure in some patients. Because of the small number of available studies, it was not possible to perform subgroup analyses in this meta-analysis, but future studies with larger samples may prove a significant benefit only for those patients with prior ERCP failure.

Regarding the type of lithotripsy used, two studies^4,10^ used laser lithotripsy, while the other^18^ electrohydraulic lithotripsy. Previous prospective studies report a very high success rate in the clearance of the bile duct with laser lithotripsy, with rates varying between 79% and 97%^31^ ^,^ ^49^ Veld et al^71^. recently published a systematic review comparing all types of lithotripsy (laser, electrohydraulic and extracorporeal) in the treatment of difficult biliary lithiasis following failure of ERCP. In their study, laser lithotripsy presented a 95% biliary clearance rate (EHL 88% and ESWL 84% (extracorporeal shock wave lithotripsy), with a general morbidity rate of 10%, EHL 13% and ESWL 8% and no reported mortality. From that data, we can conclude that laser lithotripsy is the most successful treatment for difficult choledocholithiasis. However, the results of our meta-analysis suggest that the electrohydraulic lithotripsy technique is superior than laser lithotripsy, with statistical significance.

This discrepancy between the results in our study and the results reported by Veld et al^71^ can be explained. These authors in the analysis of different lithotripsy techniques included articles that did lithotripsy under fluoroscopic guidance and not cholangioscopy^31^ ^,^ ^36^ ^,^ ^41^ ^,^ ^45^ ^,^ ^71^. They also included articles in which the authors did not report whether they used fluoroscopy or POC^13^ ^,^ ^54^ **.** Additionally, one study used two alternative methods for lithotripsy, however it did not state how many of the 26 patients used EHL and how many used LL^16^. Finally, another study included lithotripsy under percutaneous or intraoperative cholangioscopy guidance (trans T-tube or transcystic)^64^. For this reason, these studies were not included in our subgroup analysis comparing EHL vs. LL. We believe that this was the etiology of the heterogeneity of the results between our studies because our analysis verified the advantage of the EHL method. We believe that this is important because it underlines the disparities that exist in the current literature on this topic.

It is important to also note that in the clinical trials conducted by Buxbaum et al^10^. and Franzini et al.^18^, the procedures were performed by endoscopists with extensive experience in ERCP but with limited experience using cholangioscopy**.** On the other hand, Angsuwatcharakon et al. ^4^ conducted their study years after the spyglass system was released and the operators already had some initial experience with the platform. Studies have shown that operator experience is related to better performance and reduction of adverse events^19^ ^,^ ^56^ **.** Therefore, possible differences in the experience of the clinicians performing the procedure between the articles could also explain the differences found in their individual results.

In the reference of the analysis of the observational studies, the mean removal rate of cholangioscopy was 88.29% with 72.7% of successful extractions with only one lithotripsy session. These levels of success are very significant compared to those of conventional methods: there is a difference of almost 10 to 20% with ERCP success rates in difficult biliary stones (the probability of success is in the range of 68% to 79%)^20^. The number of cholangioscopies required to complete the removal was on average 1.5±0.1 and on average required 47.50±6 min of procedure time per session. The total adverse event rate was of 8.7%, with 0.5% of severe adverse events (assessed by ASGE lexicon, Cotton et al., 2010)^15^, similar to findings in the literature for conventional ERCP (serious adverse event rate 1%, CI95: 1%-2%)^2^ ^,^ ^15^. The analysis of the observational studies have shown that the use of POCS for difficult lithiasis has a high success rate for stone removal and that it is effective and safe for extraction in patients with difficult biliary lithiasis. However, considering the success rate of cholangioscopy, there are still approximately 10% of patients who do not achieve adequate stone removal by this method. Therefore, we recommend that these patients be treated at centers of excellence.

Our study has some limitations. The first is related to the lack of data found in the literature: a limited number of RCTs exist and each had a small sample size. Another limitation was that the comparative analysis showed high heterogeneity. Using the funnel plot method, we verified the presence of some outliers, which had to be removed, treating the heterogeneity and homogenizing the sample, increasing the strength of the evidence of our study. Additionally, two of the three randomized patients used the old cholangioscope system, which may have reduced the efficacy of cholangioscopy. It was also not possible to carry out cost analysis for the procedures, due to the concealment of this information in the articles analyzed. Finally, the quality of the evidence is still affected by the fact that one of the authors did an analysis of protocol intent, in addition to not performing blinded allocation, which reduces, in some way, the reliability of our results. 

The limitations to the analysis of the observational articles included the heterogeneity due to the use of different types of cholangioscopes, equipment and accessories for lithotripsy, and variability of the operators and their skill level. However, we believe that the inclusion of many procedures performed in several different centers can more accurately express the practical and more generalizable results of lithotripsy outside of the centers we evaluated. Despite these limitations, we believe that this is the best evidence available in the literature to support the use of cholangioscopy-guided lithotripsy.

 We believe that future studies should focus on patients who had a previous attempt at stone removal and failed and that use the unique digital version of cholangioscope with electrohydraulic lithotripsy. Addition controlled studies need to be conducted and should compare hydraulic and laser lithotripsy, thus providing robust and direct evidence to corroborate our indirect comparison. With increase inoperator experience using the digital version of the cholangioscope, and by only evaluating patients with failure to remove stones on prior ERCP attempt, it is possible that these studies will show the superiority of lithotripsy by POC. Based on the results found in our meta-analysis and considering the current cost of cholangioscopy, we suggest that the POC-guided lithotripsy should only be used for the treatment of difficult bile duct stones in cases where conventional techniques fail. 

## CONCLUSION

Based on the results of our systematic review and meta-analysis, in terms of therapeutic success, mean fluoroscopy time and adverse event rate there was no statistical difference in the comparison between POC and standard ERCP endoscopic methods in the management of difficult bile duct stones. However, conventional ERCP therapies were associated with less procedural time. In patients who had a prior ERCP with failure to remove stones, our analysis verified the benefit of cholangioscopy in the therapeutic success for the clearance of stones in the common bile duct. Considering the current cost of cholangioscopy, we believe that POP-guided lithotripsy should primarily be used in cases when the conventional technique failed to initially remove stones within the common bile duct. 
